# Antimicrobial Activity of Resveratrol Analogues

**DOI:** 10.3390/molecules19067679

**Published:** 2014-06-10

**Authors:** Malik Chalal, Agnès Klinguer, Abdelwahad Echairi, Philippe Meunier, Dominique Vervandier-Fasseur, Marielle Adrian

**Affiliations:** 1 Université de Bourgogne, UMR1347 Agroécologie, ERL CNRS 6300, BP 86510, 21065 Dijon Cedex, France; E-Mail: malik.chalal@univ-reims.fr; 2 INRA, UMR1347 Agroécologie, ERL CNRS 6300, BP 86510, 21065 Dijon Cedex, France; E-Mail: agnes.klinguer@dijon.inra.fr; 3 Welience, Maison Régionale de L’Innovation, 64 A rue de Sully, CS 77124, 21071 Dijon Cedex, France; E-Mail: abdelwahad.echairi@welience.com; 4 Institut de Chimie Moléculaire de l’Université de Bourgogne, ICMUB-UMR CNRS 6302, 9 Avenue Alain Savary, 21000 Dijon, France; E-Mails: Philippe.Meunier@u-bourgogne.fr (P.M.); Dominique.Vervandier-Fasseur@u-bourgogne.fr (D.V.-F.)

**Keywords:** resveratrol, stilbenes, grapevine, downy mildew, grey mold, *Plasmopara viticola*, *Botrytis cinerea*

## Abstract

Stilbenes, especially resveratrol and its derivatives, have become famous for their positive effects on a wide range of medical disorders, as indicated by a huge number of published studies. A less investigated area of research is their antimicrobial properties. A series of 13 *trans*-resveratrol analogues was synthesized via Wittig or Heck reactions, and their antimicrobial activity assessed on two different grapevine pathogens responsible for severe diseases in the vineyard. The entire series, together with resveratrol, was first evaluated on the zoospore mobility and sporulation level of *Plasmopara viticola* (the oomycete responsible for downy mildew). Stilbenes displayed a spectrum of activity ranging from low to high. Six of them, including the most active ones, were subsequently tested on the development of *Botrytis cinerea* (fungus responsible for grey mold). The results obtained allowed us to identify the most active stilbenes against both grapevine pathogens, to compare the antimicrobial activity of the evaluated series of stilbenes, and to discuss the relationship between their chemical structure (number and position of methoxy and hydroxy groups) and antimicrobial activity.

## 1. Introduction

Plants possess an innate immune system that prevents their infection by most of microorganisms such as oomycetes and fungi [1]. This self-defense potential includes the production of the secondary metabolites phytoalexins, antimicrobial compounds synthesized and accumulated in response to biotic or abiotic stresses [2,3]. In grapevines, they are stilbenes synthesized via the phenylalanine/polymalonate pathway [4]. The key compound resveratrol (3,5,4'-trihydroxystilbene) is formed by condensation of one molecule of *p*-coumaroyl-CoA and three molecules of malonyl-CoA by stilbene synthase (EC 2.3.1.95). Subsequent glycosylation, methoxylation or dimerization reactions provides a spectrum of resveratrol derivatives [5]. Such modifications are essential for the biological activity of the so- formed compounds [6,7,8].

A huge number of works has reported the role of stilbenes, especially resveratrol, in human health. They have attracted attention for their high preventive or curative effects on a wide range of medical disorders and are known as cardioprotective, antitumor, neuroprotective and antioxidant agents (for reviews, see [9,10,11,12,13]). In comparison, the antimicrobial properties of stilbenes have been less investigated. In grapevines, stilbenes are constitutively accumulated at high concentrations in the heartwood where they act as phytoanticipins and can prevent the development of wood decay [14,15]. In other tissues, they are accumulated in response to various microorganisms including pathogens: *Plasmopara viticola*, *Erysiphe necator*, *Botrytis cinerea*, *Phaeomoniella chlamydospora*, *Fusarium solani*, *Cladosporium cucumerinum*, *Pyricularia oryzae*, *Aspergilli*, *Rhizopus stolonifer* (for review, see [5,16,17]). Whereas resveratrol generally shows a moderate antimicrobial activity, it is the precursor of more active derivatives such as pterostilbene and viniferins (for reviews, see [5,16,17]).

Recently, interest in the bioproduction and chemical synthesis of stilbenes has emerged to identify highly active molecules that could be used for medical applications and/or plant disease control [18,19,20,21,22]. Previous studies have shown that resveratrol is not the most active stilbene regarding antimicrobial activity [5,6,16,17]. In this study, 13 *trans*-resveratrol analogues were synthesized as previously described [20] to identify better candidates than *trans*-resveratrol to control two harmful grapevine pathogens: *P. viticola* (downy mildew) and *B. cinerea* (grey mold). This allowed us to compare their antimicrobial activity against both pathogens and to discuss the chemical structure/antimicrobial activity relationships of these compounds.

## 2. Results and Discussion

### 2.1. Activity of Stilbenes on P. viticola

**Figure 1:**
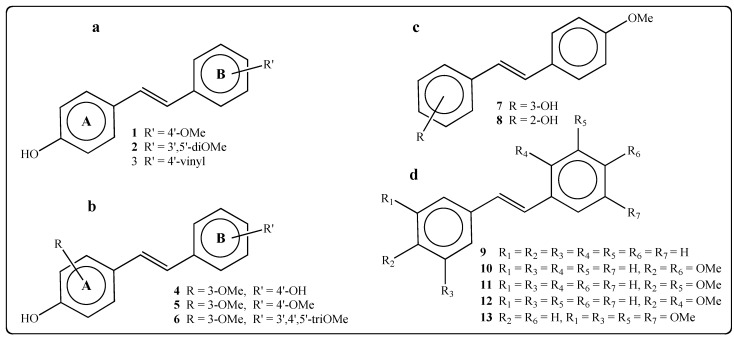
Structure of the stilbenes used for bioassays. (**a**) 4-OH stilbenes bearing substituents on cycle B; (**b**) 4-OH stilbenes bearing substituents on cycle A and/or cycle B; (**c**) Structure of 2-OH and 3-OH stilbenes; (**d**) Structure of stilbenes without phenolic function.

A series of 13 *trans*-resveratrol analogues was synthesized via Wittig or Heck reactions and the structures were confirmed by ^1^H, ^13^C-NMR, HRMS, and IR after purification [20]. The compounds differed by the number and position of hydroxy and/or methoxy groups (Figure 1).

**Figure 2:**
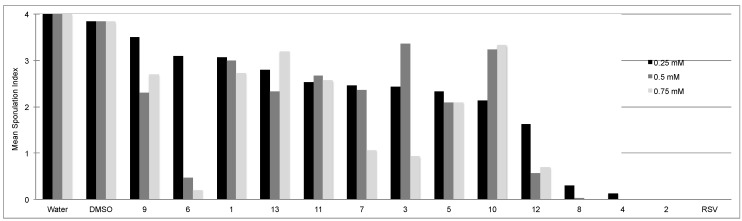
.Effect of stilbenes (0.25, 0.5 and 0.75 mM) on *P. viticola* sporulation*.* Leaf disks (10/condition) were inoculated with a *P. viticola* sporangia suspension added by the stilbenes, water or DMSO (2% v/v final concentration) as controls. The index of sporulation was scored at 6 days post-inoculation on a scale of 0 to 4, where 0 = no visible sporulation, 1 = 1% to 25%, 2 = 26% to 50%, 3 = 51% to 75%, and 4 = 76% to 100% of the disk area covered. Values represent the mean index from three independent experiments.

Their activity was first assessed in parallel with resveratrol against *P. viticola*, the oomycete responsible for grapevine downy mildew. This pathogen is obligatory and biotrophic *i.e.*, it develops in leaving grapevine tissues and cannot be grown on artificial media. The assays were therefore performed by adding the compounds to a sporangia suspension (0.25, 0.5 and 0.75 mM) prior to inoculation of leaf disks. The level of sporulation was determined by visual scoring at 6 days post inoculation as previously described [23]. Stilbenes were prepared in DMSO to ensure their dissolution and the final DMSO concentration in suspensions was 2% (v/v). As there was interest in identifying stilbenes more active than resveratrol, the three concentrations assessed for this bioassay were chosen on the basis of resveratrol ID_100_ values towards *P. viticola* and/or *B. cinerea* previously reported in the literature [24,25,26]. The results obtained are presented in Figure 2.

DMSO alone only slightly reduced the level of sporulation in comparison with water control. All resveratrol analogues showed activity compared to both water and DMSO controls. The most active ones were **2**, **4**, **8**, and resveratrol (**RSV**); especially **2** and **RSV** that totally inhibited the sporulation at all concentrations assessed. Compounds **6** and **12** were also highly active at 0.5 and 0.75 mM and, to a lesser extent, compounds **3** and **7** at 0.75 mM. Compound **2** corresponds to pterostilbene, previously reported as having the highest antimicrobial activity among natural resveratrol derivatives [6,25,26]. In most cases, the presence of lateral groups increases the activity against *P. viticola*. For disubstituted stilbenes, the nature of the groups (-OH and -OMe *vs.* -OMe and -OMe) is important for activity although their position looks essential (comparisons **1** *vs.* **10**, **7** *vs.* **11**, **8** *vs.* **12**). The presence of a methoxy group in position 4’ together with a methoxy or hydroxy group in position 2 confer high activity. Resveratrol and tri-substituted derivatives were the most active compounds, suggesting the importance of the hydroxy group at position 4'. The presence of more than three groups does not increase activity.

A prerequisite for successful downy mildew infection is zoospore mobility. Hence, once released by sporangia, bi-flagellated zoospores have to swim into water droplets present at the leaf surface to reach stomata where they encyst and form a germ tube that uses these natural pores to enter the leaf. The effects of the most active stilbenes were therefore measured on zoospore mobility (Table 1).

**Table 1:** Effects of stilbenes (**RSV** and compounds **2**, **4**, **6** and **8**) on the mobility of *P. viticola* zoospores. Values correspond to the percentage of mobile zoospores released by sporangia in a suspension added by stilbenes (0.25, 0.5, and 0.75 mM). The value of 100 was attributed to the number of mobile zoospores determined in the water control.

Compound	0.25 mM	0.5 mM	0.75 mM
2	0	0	0
8	0	0	0
RSV	38	0	0
46	3846	2315	00

For these bioassays, DMSO slightly reduced the mobility of zoospores (90% of mobile zoospores towards the water control) and the most active compounds were **2** and **8**. Curiously, no mobile zoospores could be observed in the presence of compound **8** whereas sporulation occured at 0.25 mM and, to a lesser extent, at 0.5 mM. The mobility of zoospores in these conditions is maybe too low to be measured but sufficient to allow few zoospores to reach stomata and initiate infection. The opposite was observed for **RSV** at 0.25 mM for which mobile zoospores were observed whereas no sporulation could be detected. Mobile zoospores might reach stomata but they could not germinate. The effects of natural stilbenes on the mobility of zoospores have been previously reported [26]. Resveratrol was less active than pterostilbene, as in our conditions, and also than viniferins.

### 2.2. Activity of Stilbenes on B. cinerea

We were interested in identifying stilbenes with high antimicrobial activity against both grapevine pathogens. On the basis of the results obtained with *P. viticola*, compounds **2**, **4**, **6**, and **8** were evaluated against *B. cinerea*, the necrotroph fungus responsible for grey mold. Compounds **1** and **7** were included to allow comparison with **8**. Conidia suspensions were prepared in the culture medium and added by stilbenes or DMSO (2% v/v final concentration) as control. Bioassays were performed in microplates and the mycelium development was automatically recorded at regular time intervals by spectrophotometry for 60 h. Stilbenes were tested at concentrations ranging from 0.01 to 0.75 mM to allow the determination of their IC_50_ value (Table 2). As previous studies have clearly shown that resveratrol is less active than pterostilbene (**2**) against *B. cinerea* [5,6,16,17,25] it was not included in these assays at different concentrations but only at 0.5 mM as positive control. The IC_50_ of pterostilbene was therefore used for comparison. Resveratrol (0.5 mM) was systematically included in each bioassay as reference.

**Table 2:** Effects of stilbenes on *B. cinerea* development. Values correspond to the concentration that inhibits 50% of the mycelial growth (IC_50_). SE: Standard error.

Compound	IC_50_ ± SE (µM)
7	28 ± 3
8	30 ± 5
2	52 ± 4
4	55 ± 11
1	>100
6	>100

DMSO alone did not inhibit the mycelial development (data not shown). Compound **8** appeared highly interesting since it showed high antimicrobial activity against both pathogens. It was more efficient than pterostilbene (**2**), generally described as the most active resveratrol analogue, on *B. cinerea*. This highlights the interest of this disubstituted stilbene. According to the results obtained with *P. viticola*, compound **4** also showed a high activity whereas compounds **1** and **6** were less active. Interestingly, compound **7** showed a high antimicrobial activity on *B. cinerea* but was less effective on *P. viticola*. Altogether, these results confirm that the chemical structure of stilbenes is essential for their biological activity. As example, resveratrol methylation confers a higher antifungal activity [6,19,25]. Monoglucosides also exhibit an antioxidant activity that depends on the location of the hydroxy groups and the type of the sugar residue [7]. Albert *et al.* [19] also reported this structure/biological activity relationship. However, in some cases, it depends on microorganisms. As example, they observed that the activity of methoxylated stilbenes was significant for fungi but low for bacteria. It would be interesting to determine what make the specificity of the activity of such compounds.

**Figure 3:**
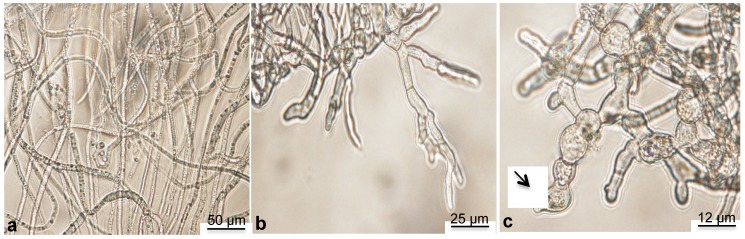
.Representative photographs showing *B. cinerea* mycelial development (**a**) in the culture medium alone as control or (**b**,**c**) added by compounds **2**, **4**, **7** or **8**. Observations were made using a Leitz DMRB microscope.

At the end of the experiments, the fungus was collected from the microplates and observed by microscopy. Characteristic images are presented in Figure 3. In control conditions, *B. cinerea* developed a thick network of long hyphae (Figure 3a). Conversely, in presence of active compounds (**2**, **4**, **7** and **8**), the mycelium development was restricted (Figure 3b). One could observe short gem tubes and thinned hyphal tips, indicating that the fungal development was stopped. In some cases, dead conidia released their intercellular content (Figure 3c, arrow). These observations are typical of the toxic effects of stilbenes [25,27].

## 3. Experimental

### 3.1. Synthesis

The syntheses and characterization of the 13 stilbenes assessed were previously reported [20]. Briefly, preparation of compounds **4**–**8**, and **10**–**12** was achieved using palladium catalysis Heck coupling reactions. Stilbenes **1**–**3**, **10** and **13** were prepared through classic Wittig reactions.

### 3.2. Plant Material

Grapevine (*V. vinifera* L. cv. Marselan) herbaceous cuttings were grown in a glasshouse at a temperature of 24 and 18 °C (day and night, respectively), with a photoperiod of 16 h light, and at a relative humidity (RH) of 70% ± 10%, as previously described [28]. Once they developed six leaves, the second and third leaves below the apex were excised and used for *P. viticola* inoculation.

### 3.3. P. viticola Bioassays

#### 3.3.1. Assessment of Stilbene Effects on Sporulation

The *P. viticola* strain was maintained on Marselan plants as previously described [28]. For bioassays, leaf disks (1 cm diameter) were punched out and placed in a Petri dish on wet filter paper. They were inoculated with 30 μL of a 10^5^ sporangia/mL suspension prepared in distilled water and added by stilbenes dissolved in DMSO (0.25, 0.5 and 0.75 mM, 2% v/v final concentration of DMSO). The suspension was also added by water and DMSO alone (2% v/v final concentration) as controls. The antimicrobial activity of compounds was determined 6 days post inoculation (dpi) by visual scoring of the index of sporulation on a scale of 0 to 4, where 0 = no visible sporulation, 1 = 1% to 25%, 2 = 26% to 50%, 3 = 51% to 75%, and 4 = 76% to 100% of the disk area covered. Ten leaf disks were prepared per condition and the experiment was repeated three times.

#### 3.3.2. Assessment of Stilbene Effects on Zoospore Mobility

Assays were performed using a modified method previously described [26]. A sporangia suspension of *P. viticola* was prepared at 5 × 10^4^ sporangia·mL^−1^ in distilled water and added by stilbene dissolved in DMSO (0.25, 0.5 and 0.75 mM, 2% v/v final concentration of DMSO). The suspension was also added by water and DMSO (2% v/v final concentration) alone as controls. After 45 min at room temperature with frequent and gentle handly stirring to allow the release of zoospores, 30 µL of the suspension was deposited on a Malassez cell. The number of zoospores passing through an area of one rectangle of the cell was determined during 1 min using a microscope (magnification ×40).

### 3.4. B. cinerea Bioassays

A conidial suspension of the *B. cinerea* strain BMM was prepared at 2 × 10^5^ conidia/mL of PDB (Potato Dextrose Broth 1/4) medium. Stilbenes prepared in DMSO were added to the suspension (at 0.01, 0.02, 0.05, 0.1, 0.25 and 0.5 mM, 2% v/v final concentration of DMSO). The suspension was also added by DMSO alone (2% v/v final concentration) as control. One hundred microliters of each suspension were distributed in microplates for automatic spectrophotometry recording (Bioscreen C, Thermoelectron Led, St Herblain, France). The absorbance at 492 nm was measured and recorded every 2 h for 60 h.

## 4. Conclusions

This study allowed us to compare the antimicrobial activity of 13 *E-*stilbenes with resveratrol on two grapevine pathogens. Altogether, the results confirm the importance of the chemical structure of stilbenes regarding their biological activity. However, they did not allow us to draw a clear and direct relationship between the structure of a compound (number/position of OH- and OCH_3_- groups) and its antimicrobial activity, making it difficult to predict the level of antimicrobial activity of a stilbene. The results also highlight that the activity of resveratrol analogues is microorganism dependent. Among the 13 *trans*-resveratrol derivatives assessed, only three of them were highly active against both pathogens. One of them is the already known pterostilbene. The other ones are 2-hydroxy, 4-methoxystilbene (8) and 3-methoxy-4,4'-hydroxystilbene (4). Compound **7** (3-hydroxy-4'-methoxystilbene) also looks interesting, but only against *B. cinerea*. Interestingly, compounds **7** and **8** showed a higher antimicrobial activity against *B. cinerea*, compared to the highly active pterostilbene. It would be interesting to investigate the biological activity of these new compounds against a larger spectrum of microorganisms.
